# Supplementary material for: Move it or lose it: interspecific variation in risk response of pond-breeding anurans
Source: PeerJ. 2019 Jun 7;7:e6956. doi: 10.7717/peerj.6956 (PMC6557263; doi:10.7717/peerj.6956)
Supplement: Supplemental Information 1 [file peerj-07-6956-s001.docx]

Table 1S: Parameter estimates of generalized linear model.

|  |  | Parameter estimate |
| --- | --- | --- |
| Species | *L. sphenocephalus* | 0.000 |
|  | *A. blanchardi* | 0.627 |
|  | *H. cinerea* | -4.961 |
| Location | Middle | 0.000 |
|  | Edge | 0.841 |
|  | Boundary | 0.847 |
| Month | June | -0.694 |
|  | July | -0.244 |
|  | August | -0.839 |
|  | September | 0.000 |
|  | October | -0.002 |
|  | November | 0.077 |
